# Gender disparity in the associations of overweight/obesity with occupational activity, transport to/from work, leisure-time physical activity, and leisure-time spent sitting in working adults: A cross-sectional study

**DOI:** 10.1016/j.je.2016.08.019

**Published:** 2017-08-01

**Authors:** Chun-Xiao Xu, Hong-Hong Zhu, Le Fang, Ru-Ying Hu, Hao Wang, Ming-Bin Liang, Jie Zhang, Feng Lu, Qin-Fang He, Li-Xin Wang, Xiang-Yu Chen, Xiao-Fu Du, Min Yu, Jie-Ming Zhong

**Affiliations:** a Department of Chronic Non-Communicable Diseases Control and Prevention, Zhejiang Provincial Center for Disease Control and Prevention, Hangzhou, Zhejiang, China; b Preventive Medicine Institute, Louisiana, MO, USA

**Keywords:** Overweight/obesity, Occupational activity, Transport to/from work, Leisure-time physical activity, Leisure-time spent sitting

## Abstract

**Background:**

The associations of occupational activity (OA), commuting, leisure-time physical activity (LTPA), and sitting with overweight/obesity in working adults are controversial. This study explored these factors with the risk of overall and abdominal overweight/obesity in a Chinese working population and whether these associations differ by gender.

**Methods:**

A cross-sectional study was conducted. Data analysis was done among 6739 employed participants. Multivariate logistic regression was used to estimate the odds ratios (ORs) and 95% confidence intervals (CIs) for the studied associations.

**Results:**

For male employees, those with heavy OA had a lower overall (OR 0.76; 95% CI, 0.62–0.93) and abdominal (OR 0.76; 95% CI, 0.62–0.93) overweight/obesity risk than those with light OA. Those with LTPA ≥150 min/week had a lower risk of overall (OR 0.73; 95% CI, 0.56–0.96) and abdominal (OR 0.70; 95% CI, 0.53–0.91) overweight/obesity than those with LTPA <150 min/week. Men with leisure-sitting time <2.5 h/day had a significantly lower risk of abdominal overweight/obesity than those sitting ≥4 h/day (OR 0.80; 95% CI, 0.65–0.99). And men who cycled to/from work had a lower risk of overall (OR 0.69; 95% CI, 0.53–0.90) and abdominal overweight/obesity (OR 0.71; 95% CI, 0.54–0.92) than passive transports. However, the above significant associations disappeared among female employees.

**Conclusions:**

Heavy OA, cycling to/from work, and LTPA were associated with lower risk of overall or abdominal overweight/obesity in male employees. Reducing leisure sitting time can also help male employees reduce the risk of abdominal overweight/obesity. More research on gender disparity in the risk of overweight and obesity should be done.

## Highlights

• Physical activity and sitting with overweight/obesity in employees were studied.
• Heavy occupational activity had a lower overweight/obesity risk.
• Active LTPA and cycling to work were associated with lower overweight/obesity risk.
• Reducing leisure sitting time can reduce the risk of abdominal overweight/obesity.
• The significant results were observed in male employees but not in females.

## Introduction

The overweight and obesity epidemic during the past two decades has been a significant global health problem. According to the China Health and Nutrition Surveys (CHNS), the prevalence of overweight, overall obesity, and abdominal obesity among Chinese adults has greatly increased during the past 17 years.^1^ Obesity is a major risk factor for cardiovascular disease (CVD). Abdominal obesity can also be a strong predictor of obesity-related morbidity and mortality independent of body mass index (BMI), since it is considered to be more closely associated with the risk of morbidity and mortality than overall obesity.^2^

For prevention purposes, it is important to identify factors to prevent overall and abdominal overweight/obesity in the population. Obesity is mainly attributed to imbalance of higher energy intake than energy expenditure. Promotion of physical activity is thus proposed as a strategy for increasing energy expenditure. The associations of certain aspects of physical activity, such as occupational activity (OA), transportation, and leisure-time physical activity (LTPA) with obesity, however, have been inconsistent in epidemiologic studies among different populations. For example, some studies show a negative association^3^, ^4^ between OA and obesity, whereas others show a positive association^5^ or no association at all.^6^, ^7^ Some report a negative association between active transportation and adiposity,^8^, ^9^ whereas others report associations in the opposite direction^10^, ^11^ or no associations.^12^

Other factors, such as prolonged sitting, have been reported to be related to obesity, metabolic syndrome, type 2 diabetes, premature mortality, and other health risks.^13^ The association of prolonged sitting with obesity and metabolic syndrome may be independent of the protective contributions of physical activity.^13^ But little is known about the relationship between OA and sedentary and active behaviors outside work. One study shows that workers in sedentary and active jobs do not differ in their sitting time or step counts outside work time.^14^

Most studies of physical activities and obesity have been conducted in western countries. Although the Chinese society is moving toward modern inactive physical lifestyles, the nature of both physical activity and leisure-sitting time may be quite different between China and western countries.^15^ Little is known about certain aspects of physical activity and leisure-time spent sitting and overall and abdominal overweight/obesity in China, especially in working adults. A better understanding of these factors for the development of overall and abdominal overweight/obesity in a Chinese working population is needed.

This cross-sectional study will examine the distributions of and the relationships between OA and workers sitting and being physically active outside work time and explore the associations of OA, transport to/from work, leisure-time spent sitting, and other physical activity with overall and abdominal overweight/obesity in a population of working adults in China and whether these associations differ by gender.

## Methods

### Study design and participants

A large-scale population-based cross-sectional study was conducted to estimate the prevalence and to identify potential risk factors of metabolic syndrome in Zhejiang Province between 2009 and 2010. Multi-stage stratified cluster sampling method was used to select the study participants. Sampling methods used in this survey have been published elsewhere.^16^ A total of 19,113 individuals were invited to participate in the study. In total, 17,434 participants (8169 males and 9265 females) were enrolled in the survey. All participants had no history of cancer or mental illness, were not receiving any medication, and were aged 18 years or older at the time of enrollment. Employment status was categorized into full-time, part-time, and unemployment based on a self-reported questionnaire. In this study, we focused our analyses on 6739 workers who reported working full time and were aged 18–69 years (3797 men and 2942 women), after exclusion of part-time, unemployed, and participants with missing data on employment.

Signed written consent forms were collected from all participants, and the study protocol was approved by the Ethics Committee of Zhejiang Provincial Center for Disease Control and Prevention.

### Anthropometric measurements

The survey was conducted indoors and face-to-face using a structured questionnaire. The questionnaire included two parts: a brief profile of the whole family, such as annual household income, and assessment of demographic characteristics (e.g., age, gender, educational level, and marital status) and lifestyle factors (e.g., smoking, alcohol intake, physical activity, and dietary habits). Height, weight, and waist circumference (WC) in centimeters were measured while the participants were dressed with light clothing without shoes after overnight fasting. WC was measured three times, and the mean value was adopted. BMI was calculated as weight in kilograms divided by height in meters squared (kg/m^2^).

### Definitions of outcome, exposures, and other adjusted factors

Overall and abdominal overweight/obesity were defined based on the criteria of the Working Group on Obesity in China (WGOC)^17^: overall overweight was defined as BMI 24–28.0 kg/m^2^, and overall obesity was defined as BMI ≥28.0 kg/m^2^. Abdominal overweight and obesity were defined sex-specifically: WC 80–89 cm and WC ≥ 90 cm for women, and WC 85–94 cm and WC ≥ 95 cm for men, respectively.

To ensure comparability with other studies, our study also incorporated the criteria defined by the World Health Organization (WHO) for Asians^18^: overall overweight was defined as BMI 23–27.5 kg/m^2^, and obesity was defined as BMI ≥27.5 kg/m^2^.

District economic level were classified into five groups^16^: type 1 urban district (highest level), type 2 urban districts, type 1 rural county, type 2 rural county, and type 3 rural county (lowest level).

Food frequency and quantitative intake was assessed by administering a questionnaire composed of questions regarding 144 food items. According to the caloric value of each item per 50 g, daily total caloric intake was calculated.^16^

LTPA was reported as times per day and the number of days and duration in hours and or minutes per episode of any physical activity for the purpose of recreation and/or fitness, such as leisure walking, playing basketball, and swimming in a typical week.^19^ LTPA was categorized at a cut-off point <150 min/week or ≥150 min/week.

The participants reported their OA according to the following three categories^20^: ‘light’ OA, defined as physically very easy, mostly sitting office work (e.g., a secretary); ‘moderate’, defined as work mostly standing and walking (e.g., a store assistant or light industrial worker); and ‘heavy’, defined as work like lifting or heavy manual labor (e.g., an industrial worker). A detailed description of the questions has been published elsewhere,^21^, ^22^ and questions were constructed and evaluated previously.^23^

Transport to/from work included walking, bicycle, electromobile, motorcycle, car, bus, train, and working at home. The respondents were asked about their modes of transportation to/from work, which were determined by response to the question, “What do you usually use as a transport to/from work?”. Similar transport to/from work questions have been used in other studies.^24^ Walking and bicycling were classified as active commuting. Traveling by electromobile, motorcycle, car, bus, or train, and working at home were classified as passive commuting.

The activities of leisure-time spent sitting included watching television, reading, using a computer outside work time, playing poker or mahjong, and others. All participants reported the time in hours and or minutes they spent sitting during leisure-time in a usual weekday. Similar leisure-time sitting questions have been used in other studies.^19^, ^25^ Leisure-time spent sitting was categorized into <2.5 h/day, 2.5 to <4 h/day, and ≥4 h/day.

### Statistical analyses

Logistic regression were used to estimate odds ratios (ORs) and 95% confidence intervals (CIs) for the associations between overall and abdominal overweight/obesity and OA, transport to/from work, LTPA, and leisure-time spent sitting. Adjusted factors were age (continuous), gender (categorical), education (categorical), daily total calorie intake (continuous), smoking (categorical), drinking (categorical), and district economic level (categorical). Multivariate logistic analyses were conducted to estimate the associations of overall and abdominal overweight/obesity with certain type of physical activity, after adjusting for other factors, including mutual adjustment of the other physical activities. Multivariate logistic analysis was used to estimate the associations of overall and abdominal overweight/obesity with leisure-time spent sitting, after adjusting for other factors, including physical activities (OA, transport to/from work, and LTPA). The analyses were stratified by gender. The interaction between gender and physical activity or leisure-time sitting on the risk of overweight/obesity was also evaluated. All the main effects and interactions were entered in the model simultaneously.

All tests were two-sided, and the statistical significance was set at *P* < 0.05. All statistical analyses were performed with SPSS for Windows 19.0 (SPSS Inc., Chicago, IL, USA).

## Results

The distributions of age, education, district economic level, daily total caloric intake, OA, LTPA, leisure-time sitting, BMI, waist circumference, overweight/obesity, and abdominal overweight/obesity are shown in Table 1. Of the 6739 full-time employed participants (mean age: 41.3; standard deviation, 11.4 years) included in the analysis, 3797 (56.3%) were men. A total of 44.1% of men reported moderate OA, and 55.5% of women reported light OA. The proportion of active transportation in women (25.7%) was significantly greater than that in men (16.1%). The proportion of LTPA and leisure sitting time was similar between men and women. Significant inter-gender differences were observed in the proportion of overall overweight/obesity: 42.3% of men compared with 30.8% of women using WGOC's definition (*P* < 0.01) or 54.5% of men compared with 41.2% of women using WHO's definition (*P* < 0.01). Significant inter-gender differences were also observed in the proportion of abdominal overweight/obesity: 43.7% of men compared with 36.0% of women (*P* < 0.01).

**Table 1:** Characteristics of working adults in the 2009–2010 Zhejiang Metabolic Syndrome Prevalence Survey, China.

Characteristics	Total n = 6739	Men n = 3797	Women n = 2942	*P* value
Age, years	41.3 (11.4)	42.7 (11.6)	39.5 (10.9)	<0.01
Age group, years, n (%)				<0.01
18–24	538 (8.0)	266 (7.0)	272 (9.2)	
25–34	1540 (22.9)	782 (20.6)	758 (25.8)	
35–44	1946 (28.9)	1006 (26.5)	940 (32.0)	
45–54	1758 (26.1)	1064 (28.0)	694 (23.6)	
≥55	957 (14.2)	679 (17.9)	278 (9.4)	
Education, n (%)				<0.01
Primary or lower	2081 (30.9)	1141 (30.0)	940 (32.0)	
Secondary	2848 (42.3)	1654 (43.6)	1194 (40.6)	
High school/technician school	1262 (18.7)	729 (19.2)	533 (18.1)	
College and above	547 (8.1)	273 (7.2)	274 (9.3)	
Smoking, n (%)				<0.01
Non-smoker	4377 (65.0)	1533 (40.4)	2844 (96.7)	
Ex-smoker	353 (5.2)	272 (7.2)	81 (2.8)	
Current smoker	2009 (29.8)	1992 (52.4)	17 (0.5)	
Drinking, n (%)				<0.01
Non-drinker	4485 (66.6)	1791 (47.2)	2694 (91.6)	
Ex-drinker	227 (3.4)	162 (4.3)	65 (2.2)	
Current drinker	2027 (30.1)	1844 (48.6)	183 (6.2)	
District economic level, n (%)				<0.01
Type 3 rural county	1151 (17.1)	732 (19.3)	419 (14.2)	
Type 2 rural county	1861 (27.6)	1001 (26.4)	860 (29.2)	
Type 1 rural county	1236 (18.3)	673 (17.7)	563 (19.1)	
Type 2 urban districts	1481 (22.0)	831 (21.9)	650 (22.1)	
Type 1 urban districts	1010 (15.0)	560 (14.7)	450 (15.4)	
Occupation activity, n (%)				<0.01
Light	3117 (46.3)	1483 (39.0)	1634 (55.5)	
Moderate	2787 (41.4)	1674 (44.1)	1113 (37.8)	
Active	835 (12.3)	640 (16.9)	195 (6.7)	
Transport to/from work, n (%)				<0.01
Any passive	5370 (79.7)	3185 (83.9)	2185 (74.3)	
Walking	752 (11.2)	333 (8.8)	419 (14.2)	
Cycling	617 (9.1)	279 (7.3)	338 (11.5)	
Daily caloric intake,^a^ kcal/day	2037.80 (592.45)	2151.52 (618.54)	1890.91 (521.54)	<0.01
Leisure-time physical activity, n (%)				0.17
0–149 min/week	6234 (92.5)	3527 (92.9)	2707 (92.0)	
≥150 min/week	505 (7.5)	270 (7.1)	235 (8.0)	
Leisure-time sitting,^b^ n (%)				0.28
<2.5 h/day	3399 (52.9)	1904 (52.2)	1495 (53.9)	
2.5 to <4 h/day	2187 (34.1)	1250 (34.3)	937 (33.8)	
≥4 h/day	836 (13.0)	493 (13.5)	343 (12.4)	
Body mass index, kg/m^2^	23.2 ± 3.3	23.6 ± 3.2	22.7 ± 3.3	<0.01
Overweight/obese, n (%)^c^	2511 (37.3)	1605 (42.3)	906 (30.8)	<0.01
Overweight/obese, n (%)^d^	2942 (43.7)	2069 (54.5)	1211 (41.2)	<0.01
Waist circumference, cm	80.8 ± 9.6	83.4 ± 9.2	77.4 ± 9.0	<0.01
Abdominal overweight/obese, n (%)	2717 (40.3)	1658 (43.7)	1059 (36.0)	<0.01

Values reported as mean (standard deviation), unless otherwise noted.

a Not available: n = 52.

b Not available: n = 317.

c Measured by WGOC cut-offs.

d Measured by WHO cut-offs.

Table 2 shows transport to/from work, leisure-time spent sitting, and other physical activity levels by OA stratified by gender. In the multivariate analyses, after adjustment for age, educational level, daily total caloric intake, smoking, drinking, district economic level, and OA, male employees with light OA were more likely to walk or bicycle to/from work than those with heavy OA (OR 1.73; 95% CI, 1.31–2.28). Male employees were more likely to be physically active during leisure time, as with less OA (moderate OA: OR 1.79; 95% CI, 1.03–3.12; light OA: OR 3.49; 95% CI, 2.03–5.98). Female employees with light OA were more likely to be more physically active during leisure time than those with heavy OA (OR 3.80; 95% CI, 1.51–9.55). Female employees with moderate OA were more likely to sit less than 4 h per day during leisure-time (OR 1.64; 95% CI, 1.04–2.59) than those with heavy OA.

**Table 2:** Likelihood of being active by occupational activity category stratified by gender in the 2009–2010 Zhejiang Metabolic Syndrome Prevalence Survey, China.

Gender		Male							
Occupational activity		Heavy		Moderate			Light		
Physical activity Outside work	Total	n (%)	Ref	n (%)	OR^a^ (95% CI)	OR^b^ (95% CI)	n (%)	OR^a^ (95% CI)	OR^b^ (95% CI)
Transport to/from work									
Any passive	3185 (83.9)	541 (84.5)		1478 (88.3)			1166 (78.6)		
Active	612 (16.1)	99 (15.5)	1.00	196 (11.7)	0.82 (0.63–1.07)	0.91 (0.69–1.20)	317 (21.4)	**1.63 (1.27**–**2.10)**	**1.73 (1.31**–**2.28)**
Leisure-time physical activity									
0–149 min/week	3527 (92.9)	624 (97.5)		1591 (95.0)			1312 (88.5)		
≥150 min/week	270 (7.1)	16 (2.5)	1.00	83 (5.0)	**2.16 (1.25**–**3.72)**	**1.79 (1.03**–**3.12)**	171 (11.5)	**5.30 (3.14**–**8.93)**	**3.49 (2.03**–**5.98)**
Leisure-time sitting									
≥4 h/day	493 (13.5)	89 (14.2)		201 (12.4)			203 (14.5)		
<4 h/day	3154 (86.5)	537 (85.8)	1.00	1419 (87.6)	1.22 (0.93–1.60)	1.04 (0.79–1.38)	1198 (85.5)	1.01 (0.77–1.33)	0.93 (0.69–1.24)

Gender		Female							
Occupational activity		Heavy		Moderate			Light		
Physical activity Outside work	Total	n (%)	Ref	n (%)	OR^a^ (95% CI)	OR^b^ (95% CI)	n (%)	OR^a^ (95% CI)	OR^b^ (95% CI)
Transport to/from work									
Any passive	2185 (74.3)	140 (71.8)		858 (77.1)			1187 (72.6)		
Active	757 (25.7)	55 (28.2)	1.00	255 (22.9)	0.83 (0.58–1.17)	0.79 (0.55–1.14)	447 (27.4)	1.11 (0.79–1.55)	1.01 (0.70–1.44)
Leisure-time physical activity									
0–149 min/week	2707 (92.0)	188 (96.4)		1053 (94.6)			1466 (89.7)		
≥150 min/week	235 (8.0)	7 (3.6)	1.00	60 (5.4)	1.58 (0.71–3.51)	2.02 (0.78–5.20)	168 (10.3)	**3.22 (1.49**–**6.98)**	**3.80 (1.51**–**9.55)**
Leisure-time sitting									
≥4 h/day	343 (12.4)	31 (16.6)		99 (9.4)			213 (13.9)		
<4 h/day	2432 (87.6)	156 (83.4)	1.00	955 (90.6)	**2.01 (1.30**–**3.12)**	**1.64 (1.04**–**2.59)**	1321 (86.1)	1.33 (0.88–2.01)	1.30 (0.84–2.01)

a Adjusted for age.

b Adjusted for age, educational level, daily total caloric intake, smoking, drinking, district economic level and occupational activity.

Table 3 presents adjusted risk of being overweight/obese by OA, transport to/from work, LTPA, and leisure-time spent sitting stratified by gender. Based on WGOC criteria, male employees with jobs involving in heavy OA had a significantly lower risk of overall (OR 0.76; 95% CI, 0.62–0.93) or abdominal (OR 0.76; 95% CI, 0.62–0.93) overweight/obesity compared to those with light OA. Men who had LTPA ≥150 min/week had a significantly lower risk of overall (OR 0.73; 95% CI, 0.56–0.96) and abdominal overweight/obesity (OR 0.70; 95% CI, 0.53–0.91) compared to those who had LTPA <150 min/week, after adjusting for other risk factors. Men having less than 2.5 h per day leisure-time spent sitting had an independently lower risk of abdominal overweight/obesity (OR 0.80; 95% CI, 0.65–0.99) compared to those sitting 4 or more hours per day. Compared to passive transports, male employees who bicycled to/from work had lower risk of overall (OR 0.69; 95% CI, 0.53–0.90) and abdominal (OR 0.71; 95% CI, 0.54–0.92) overweight/obesity, after adjustment. However, no significant associations of physical activity and leisure spent sitting with overweight/obesity were observed among female employees (Table 4). The results for overweight/obesity defined by WHO were similar to those for overweight/obesity defined by WGOC (eTable 1).

**Table 3:** Adjusted risk of being overweight/obesity or abdominal overweight/obesity by leisure-time physical activity, leisure-time sitting, transport to/from work, and occupational activity category in male workers in the 2009–2010 Zhejiang Metabolic Syndrome Prevalence Survey, China.

Variable	Total	Overweight/obesity^a^			Abdominal overweight/obesity		
	n (%)	n (%)	OR^b^ (95% CI)	OR^c^ (95% CI)	n (%)	OR^b^ (95% CI)	OR^c^ (95% CI)
Total	3797 (100.0)	1605 (42.3)			1658 (43.7)		
Transport to/from work							
Any passive	3185 (83.9)	1352 (42.4)	1.00	1.00	1388 (43.6)	1.00	1.00
Walking	333 (8.8)	149 (44.7)	0.99 (0.79–1.25)	0.88 (0.69–1.13)	160 (48.0)	1.07 (0.85–1.34)	0.98 (0.76–1.25)
Cycling	279 (7.3)	104 (37.3)	**0.69 (0.53**–**0.89)**	**0.69 (0.53**–**0.90)**	110 (39.4)	**0.70 (0.54**–**0.90)**	**0.71 (0.54**–**0.92)**
Leisure-time physical activity							
0–149 min/week	3527 (92.9)	1463 (41.5)	1.00	1.00	1509 (42.8)	1.00	1.00
≥150 min/week	270 (7.1)	142 (52.6)	**0.67 (0.52**–**0.86)**	**0.73 (0.56**–**0.96)**^d^	149 (55.2)	**0.64 (0.50**–**0.82)**	**0.70 (0.53**–**0.91)**^f^
Leisure-time sitting							
≥4 h/day	1904 (52.2)	806 (42.3)	1.00	1.00	817 (42.9)	1.00	1.00
2.5 to <4 h/day	1250 (34.3)	512 (41.0)	0.88 (0.71–1.09)	0.91 (0.73–1.13)	529 (42.3)	0.82 (0.66–1.01)	0.86 (0.70–1.07)
<2.5 h/day	493 (13.5)	216 (43.8)	0.90 (0.74–1.10)	0.90 (0.73–1.11)	231 (46.9)	**0.81 (0.66**–**0.99)**	**0.80 (0.65**–**0.99)**
Occupational activity							
Light	1483 (39.1)	673 (45.4)	1.00	1.00	698 (47.1)	1.00	1.00
Moderate	1674 (44.1)	690 (41.2)	0.86 (0.74–1.00)	0.90 (0.77–1.05)	709 (42.4)	**0.84 (0.73**–**0.97)**	0.88 (0.76–1.03)
Heavy	640 (16.9)	242 (37.8)	**0.70 (0.58**–**0.85)**	**0.76 (0.62**–**0.93)**^e^	251 (39.2)	**0.69 (0.57**–**0.84)**	**0.76 (0.62**–**0.93)**

a Measured by WGOC cut-offs.

b Adjusted for age.

c Adjusted for age, educational level, daily total caloric intake, smoking, drinking, district economic level, occupational activity, leisure-time physical activity, leisure-time sitting, and transportation to/from work.

d Interactive OR = 0.73 (0.67–0.81), *P* < 0.001.

e Interactive OR = 0.85 (0.77–0.94), *P* = 0.002.

f Interactive OR = 0.84 (0.77–0.93), *P* = 0.001.

**Table 4:** Adjusted risk of overweight/obesity or abdominal overweight/obesity by leisure-time physical activity, leisure-time sitting, transport to/from work, and occupational activity category in female workers in the 2009–2010 Zhejiang Metabolic Syndrome Prevalence Survey, China.

Variable	Total	Overweight/obesity^a^			Abdominal overweight/obesity		
	n (%)	n (%)	OR^b^ (95% CI)	OR^c^ (95% CI)	n (%)	OR^b^ (95% CI)	OR^c^ (95% CI)
Total	2942 (100.0)	906 (30.8)			1059 (36.0)		
Transport to/from work							
Any passive	2185 (74.3)	634 (29.0)	1.00	1.00	736 (33.7)	1.00	1.00
Walking	419 (14.2)	144 (34.4)	0.98 (0.77–1.24)	0.92 (0.71–1.18)	185 (44.2)	1.16 (0.92–1.46)	1.11 (0.87–1.42)
Cycling	338 (11.5)	128 (37.9)	1.15 (0.90–1.48)	1.09 (0.84–1.41)	138 (40.8)	0.99 (0.78–1.27)	0.97 (0.75–1.25)
Leisure-time physical activity							
0–149 min/week	2707 (92.0)	820 (30.3)	1.00	1.00	960 (35.5)	1.00	1.00
≥150 min/week	235 (8.0)	86 (36.6)	0.79 (0.59–1.05)	0.77 (0.56–1.05)	99 (42.1)	0.80 (0.60–1.06)	0.85 (0.63–1.16)
Leisure-time sitting							
≥4 h/day	1495 (53.9)	477 (31.9)	1.00	1.00	547 (36.6)	1.00	1.00
2.5 to <4 h/day	937 (33.8)	291 (31.1)	1.15 (0.88–1.51)	1.14 (0.86–1.51)	330 (35.2)	0.88 (0.68–1.14)	0.95 (0.72–1.25)
<2.5 h/day	343 (12.4)	91 (26.5)	1.20 (0.90–1.59)	1.18 (0.88–1.58)	123 (35.9)	0.91 (0.69–1.19)	0.90 (0.69–1.17)
Occupational activity							
Light	1634 (55.5)	495 (30.3)	1.00	1.00	579 (35.4)	1.00	1.00
Moderate	1113 (37.8)	343 (30.8)	0.98 (0.82–1.16)	0.89 (0.74–1.07)	404 (36.3)	0.98 (0.83–1.15)	0.91 (0.81–1.16)
Heavy	195 (6.6)	68 (34.9)	1.07 (0.78–1.47)	0.99 (0.70–1.38)	76 (39.0)	0.97 (0.71–1.33)	0.97 (0.65–1.27)

a Measured by WGOC cut-offs.

b Adjusted for age.

c Adjusted for age, educational level, daily total caloric intake, smoking, drinking, district economic level, occupational activity, leisure-time physical activity, leisure-time sitting, and transportation to/from work.

To further assess whether the effect was different between men and women, we analyzed the interaction of gender with physical activity and leisure-time sitting on overweight/obesity. The association of LTPA ≥150 min/week and heavy OA with the risk of overweight/obesity among men was 0.73 (95% CI, 0.67–0.81; *P* for interaction < 0.001) and 0.85 (95% CI, 0.77–0.94; *P* for interaction = 0.002), respectively. The association of LTPA ≥150 min/week with the risk of abdominal overweight/obesity among men was 0.84 (95% CI, 0.77–0.93; *P* for interaction = 0.001) (Table 3).

## Discussion

Our study showed that heavy OA, cycling to/from work, and LTPA ≥150 min/week were significantly associated with lower risk of overall and abdominal overweight/obesity, and that short leisure-time spent sitting was significantly associated with lower risk of abdominal overweight/obesity but not with overall overweight/obesity. These significant results were observed in male employees but not in females.

Results from previous studies examining the relationship between OA and obesity are mixed. Steeves et al.^4^ and King et al.^26^ have shown that individuals having heavy OA were less likely to have overall and abdominal obesity compared with those with light OA. Ball et al.^27^ and Gutierrez-Fisac et al.^6^ have found no association. In many high-income countries, LTPA is far more prevalent than in many developing countries, including China, where OA has been a key modifiable determinant of weight gain^28^ and LTPA is relatively uncommon. Lack of physical activity in the workplace is one of the factors responsible for the prevalence of obesity. Thus, it is important to include OA in studies seeking to understand the association between physical activity and overall and abdominal overweight/obesity. Although employees having light OA were more likely to choose active transports and be physically active in leisure-time compared to those having more OA, heavy OA was significantly associated with lower risk of overall and abdominal overweight/obesity in our study. Studies have reported that LTPA can be used to prevent weight gain or obesity. Low physical activity was positively associated with obesity in our study. Consistent with the previous literature,^29^, ^30^ we discovered protective associations between LTPA intensity and overall and abdominal overweight/obesity. Moreover, the integration of physical activity into daily work life, especially for those in sedentary occupations, can have a considerable impact on reducing the burden of preventable overweight/obesity and its related diseases.

Transport to/from work can also influence obesity. Ecologic studies suggest that active commuting to work contributes to higher levels of overall individual physical activity and thus affects body weight.^31^ Walking to work could be an opportunity for physical activity for some people, with many positive health and environment outcomes. However, the absence of an association between walking to/from work and overweight and obesity in our study suggests that this type of walking may not be vigorous enough or of enough distance. The other possible explanation could be that people in China tend to walk more outside work than people in other countries, which means walking time on average is higher in Chinese, including people in the reference group. Our study suggests that cycling to/from work promote energy expenditure and significantly decrease the risk of overall and abdominal overweight/obesity in men but not in women, which is consistent with results of an Australian study.^24^ The healthy benefits of cycling are considered somewhat greater than walking because the intensity of effort is greater. Longitudinal studies have found that individuals who cycled to work had an approximately 30% reduced risk of dying.^32^, ^33^

Sedentary time is considered an important and independent risk factor in weight gain and obesity genesis.^34^ Leisure sitting time, the number of hours spent watching television, using a computer, and certain related activities, has been found to be importantly associated with overweight and high BMI.^35^, ^36^, ^37^ Our study showed short leisure sitting time was associated with a lower risk of abdominal overweight/obesity. The deleterious effect of sitting on abdominal obesity is independent of the protective effect of physical activity, after adjustment for physical activity and other risk factors. However, our results were significant among male but not among female employees. This gender disparity is consistent with results from an Australian study, which has reported that men who sat more than 6 h per day were almost twice as likely to be overweight/obese compared with men who sat for less than 45 min per day, and no association was found among women.^38^

The potential reasons for these gender-specific differences have not been fully elucidated, although several hypotheses have been proposed. For example, it has been suggested that men report higher levels of sedentary behavior than women, and men also tend to engage in different patterns of physical activity. Gender differences may also be attributed to differences in occupational or social roles.^36^ In our study, the proportion of men with heavy labor work was significantly greater than that of women. Other studies that adopted the most accurate and objective method to measure energy expenditure have also reported stronger protective associations of physical activity with BMI for men, but weaker or no associations for women.^39^, ^40^ Data regarding household physical activity, which is likely to be higher in women than men in daily life, was not available in our study and may have biased our results in the women toward the null. Recent research has highlighted the importance of including household activity in assessing total energy expenditure, primarily in women.^3^ In a cross-sectional study, however, while Lawlor et al. agreed that household activity was important in assessing sufficient levels of activity, it had no independent effect on levels of overweight in elderly white women.^41^ Moreover, the increasing availability and purchase of time-saving household devices have lead to a decline in household activity.

Our findings are limited by cross-sectional study design. Another major limitation is that the frequency, intensity, and duration of OA and commuting activity were not measured in our survey. Occupation was broadly classified into high, moderate, and low-activity levels. In addition, physical activity measures were self-reported, and the absence of household activity in our study made us unable to provide a more complete measure of physical activity. Furthermore, healthy worker effect would bias the results to the null to some extent, but such attenuation will not change our conclusion. There are a few strengths in our study. Our results have been adjusted for daily dietary intake, a factor that is rarely available in most studies for adjustment in logistic regression models. It is possible that physical activity has differential associations with food intake between men and women. That frequency, intensity, and duration of leisure-time spent sitting were collected in our survey made it possible to quantify the total volume of leisure-time spent sitting. Our study was based on a population-based survey with a relatively large sample size. Finally, our findings are consistent with and supported by other studies in different populations.

In conclusion, heavy OA, cycling to/from work, and LTPA were significantly associated with lower risk of overall and abdominal overweight/obesity in male employees. Reducing leisure-time spent sitting may prevent male employees from developing abdominal overweight/obesity. No significant results were observed in females. More research on gender disparity in the risk of overweight/obesity should be done.

## Conflicts of interest

None declared.
