# Supplementary material for: Gender disparity in the associations of overweight/obesity with occupational activity, transport to/from work, leisure-time physical activity, and leisure-time spent sitting in working adults: A cross-sectional study
Source: J Epidemiol. 2017 Aug 1;27(9):401–7. doi: 10.1016/j.je.2016.08.019 (PMC5565757; doi:10.1016/j.je.2016.08.019)
Supplement: Supplementary file 1 [file mmc1.pdf]

eTable 1. Adjusted odds ratio (OR) and 95% confidence interval (CI) of overall or abdominal overweight/obesity by leisure-time physical activity, leisure-time sitting, transport to/from work, and occupational activity category in working adults in the 2009-2010 Zhejiang Metabolic Syndrome Prevalence Survey, China

Variable	Total	Overweight/obesity[d]		
	N (%)	N (%)	OR[e] (95% CI)	OR[f] (95% CI)
Total	6739 (100.0)			
Transport to/from work				
Any passive	5370 (79.7)	2585 (78.8)	1.00	1.00
Walking	752 (11.2)	386 (11.8)	1.03 (0.88–1.21)	1.05 (0.88–1.25)
Cycling	617 (9.1)	309 (9.4)	0.92 (0.77–1.10)	0.94 (0.78–1.13)
Leisure-time physical activity				
0-149 min/week	6234 (92.5)	2992 (91.2)	1.00	1.00
≥150 min/week	505 (7.5)	288 (8.8)	**0.72 (0.60–0.87)**	**0.74 (0.60–0.91)[j]**
Leisure-time sitting				
≥4 h/day	836 (12.4)	411 (13.2)	1.00	1.00
2.5 to < 4 h/day	2187 (32.5)	1040 (33.3)	0.92 (0.79–1.08)	0.92 (0.79–1.09)
<2.5 h/day	3399 (50.4)	1669 (53.5)	0.92 (0.78–1.08)	0.92 (0.78–1.08)
Occupational activity				
Light	3117 (46.3)	1512 (46.1)	1.00	1.00
Moderate	2787 (41.4)	1351 (41.2)	0.93 (0.83–1.03)	0.91 (0.81–1.03)
Heavy	835 (12.4)	417 (12.7)	**0.82 (0.69–0.96)**	**0.83 (0.70–0.98)[k]**

[d] Measured by WHO cut-offs

[e] Adjusted for age and sex

[f] Adjusted for age, sex, educational level, daily total caloric intake, smoking, drinking, district economic level, occupational activity, leisure-time physical activity, leisure-time sitting, and transportation to/from work

[j]: Interactive OR= 0.74 (0.67-0.81)

[k]: Interactive OR= 0.83 (0.76-0.92)
